# The Evaluation of Microshear Bond Strength of Resin Cements to Titanium Using Different Surface Treatment Methods: An In Vitro Study

**DOI:** 10.3390/biomimetics7010018

**Published:** 2022-01-20

**Authors:** Mohammadreza Nakhaei, Neda Bozorgmehr, Hamidreza Rajati Haghi, Hossein Bagheri, Abdolrasoul Rangrazi

**Affiliations:** 1 Department of Prosthodontics, School of Dentistry, Mashhad University of Medical Sciences, Mashhad 9177948959, Iran; nakhaeemr@mums.ac.ir (M.N.); rajatihr@mums.ac.ir (H.R.H.); 2 School of Dentistry, Mashhad University of Medical Sciences, Mashhad 9177948959, Iran; 3 Dental Materials Research Center, Mashhad University of Medical Sciences, Mashhad 9177948959, Iran; bagherih@mums.ac.ir; 4 Dental Research Center, Mashhad University of Medical Sciences, Mashhad 9177948959, Iran

**Keywords:** hybrid abutment, titanium, resin cement, shear bond strength

## Abstract

This study attempted to investigate the effect of sandblasting and H_2_O_2_ treatments on the microshear bond strength of two commercially available resin cements. A total of 90 cube-shaped specimens of commercially pure titanium (cp-Ti) were divided into two groups of Panavia and MHA cements (*n* = 45). Samples of the Panavia group were randomly divided into three subgroups of 15 samples, including subgroups (no treatment, aluminum oxide sandblasting, and immersion in 35% hydrogen peroxide solution with halogen light). Once the treatment was completed, Panavia V5 was applied on the cp-Ti surface by a Tygon tube. The 45 specimens of the MHA cement group were randomly divided into three subgroups (*n* = 15) similarly to the Panavia group. Then, the MHA was applied on the surface of cp-Ti. A universal testing machine was used to measure and examine the microshear bond strength of cement to cp-Ti subsequent to the step of thermocycling. According to results, in the Panavia cement group, the SBS of sandblasting treatment was significantly higher than that of the H_2_O_2_ treatment subgroup (*p* < 0.05), which displayed a significantly higher SBS than that of the no-treatment subgroup (*p* < 0.001). In regard to the MHA group, the SBS of the H_2_O_2_ treatment subgroup was significantly lower than that of the sandblasting treatment subgroup (*p* < 0.001), whereas there were no significant differences between the SBS of the no treatment and H_2_O_2_ treatment subgroups (*p* = 0.35). Considering the comparison between Panavia and MHA cases, there were no significant differences observed among the no-treatment subgroups (*p* = 0.34), as well as the sandblasting treatment subgroups (*p* = 0.67), while the SBS of the H_2_O_2_ treatment subgroup in Panavia cement was higher than that of the H_2_O_2_ subgroup in MHA cement (*p* < 0.001). In conclusion, in both Panavia V5 and MHA cements, sandblasting treatment could improve the bond strength between the titanium surface. However, H_2_O_2_ treatment proved to be capable of enhancing the bond strength of Panavia V5 cement without causing any positive effects on the bond strength of MHA cement.

## 1. Introduction

Dental implants are a popular treatment option for the replacement of missing teeth in patients. The global dental implant market is expected to grow and reach USD 13 billion in 2023 [1]. In dental materials science, a dental implant can stand as a proper example for the combination of multiple disciplines, including biomechanics, tissue engineering, and surface chemistry and physics [2]. Abutment is an essential component of the dental implant prosthetic system, which is applied to join the implant crowns to dental implants [3] and has a fundamental role in performing a successful implant treatment. Although there are reports on the high survival rates of titanium abutments due to their excellent mechanical and biocompatible properties [4], its application with a thin gingival biotype results in shining a greyish hue toward the surrounding soft tissues [5]. This matter was attempted to be overcome through the introduction of hybrid abutment, which consisted of a titanium insert along with a zirconia or lithium disilicate ceramic component [6].

In hybrid abutments, the ceramic structure is attached to the titanium insert base by the usage of a resin cement. The formation of an oxide layer on the titanium may interfere with the reaction between the titanium surface and methacryloyloxydecyl dihydrogen phosphate (MDP) monomer, which would consequently affect the bond strength. In addition, this phenomenon leads to the induction of cracks and pores throughout this area, providing a suitable place for the growth and accumulation of microorganisms and plaque, and finally resulting in the occurrence of discoloration. According to the outcomes of previous studies, the microstructure and chemical properties of the surface of commercially pure titanium (cp-Ti) are important factors in the obtained bonding strength between the titanium surface and resin cements [7]. Therefore, several different surface treatments were experimented to improve the bond strength, which included sandblasting, silicoating, using functional monomers, and acid etching [8]. Nevertheless, determining the efficacy of these methods and selecting the best approach remain a considerable challenge in the fabrication of hybrid abutments.

For many years, the sandblasting technique has been applied in dentistry for increasing the mechanical adhesion between metals and adhesive resins. In the course of a blasting process, the abrasive particles are blustered onto the surface of a metal substrate through a high-velocity compressed air stream to improve the surface roughness, surface energy, and bonding surface area, as well as to cause the removal of unfavorable oxides and contaminants [9].

The chemical treatment of metal surfaces by hydrogen peroxide is recognized as another method for enhancing the obtained adhesion between metals and adhesive resin. In this procedure, Ti is immersed in a hydrogen peroxide solution to have its surface oxidized and consequently improve the bond strength [10]. The oxidation mechanism was postulated due to the formation of hydroxyl radicals and the Fenton reaction [10]. In a Fenton reaction, a metal ion takes part in the formation of a hydroxyl radical (OH) during the hydrogen peroxide (H_2_O_2_) decomposition [11]. According to related data, the application of a halogen lamp can facilitate the acceleration of Fenton reactions. In addition, previous studies have indicated that a combined treatment of hydrogen peroxide and halogen light irradiation can provide an effective surface condition with appropriate oxide film thickness, which would consequently improve the cement bond strength [12].

To obtain useful information about the effect of different surface treatments on the bond strength of resin cements, this study attempted to investigate the effect of sandblasting and H_2_O_2_ treatments on the microshear bond strength of the two commercially available resin cements. Our null hypothesis was to consider that sandblasting and H_2_O_2_ treatments have no significant effect on the microshear bond strength of the resin cements that form a bond to the titanium surface.

## 2. Materials and Methods

First, 90 cube-shaped specimens (4 × 4 × 4 mm) of commercially pure titanium (cp-Ti) (imes-icore GmbH, Eiterfeld, Germany) grade 4 was fabricated using the milling method and were then embedded in a self-cured acrylic resin (Acropars, Marlic Co. Tehran, Iran). Then, the samples were polished by silicon carbide papers (400, 600, 800, and 1000 grits) under running water. In the following, the specimens were cleaned in ethanol for 5 s and an ultrasonic bath (CD-4820, GS, Shenzhen city, Guangdong, China) in distilled water for 180 s.

In this study, we considered the application of the two resin cements of Panavia V5 (Kuraray Noritake Dental Inc., Okayama, Japan) and MultiLink Hybrid-Abutment (MHA) (Ivoclar Vivadent, Schaan, Liechtenstein) (Table 1).

Once the 90 specimens were divided into two groups of Panavia and MHA cements (*n* = 45), the samples of the Panavia group were randomly divided into three subgroups with 15 samples according to the following:

Subgroup A: The samples were only polished without any other surface treatment to undergo the application of Panavia cement.

**Figure 1:**
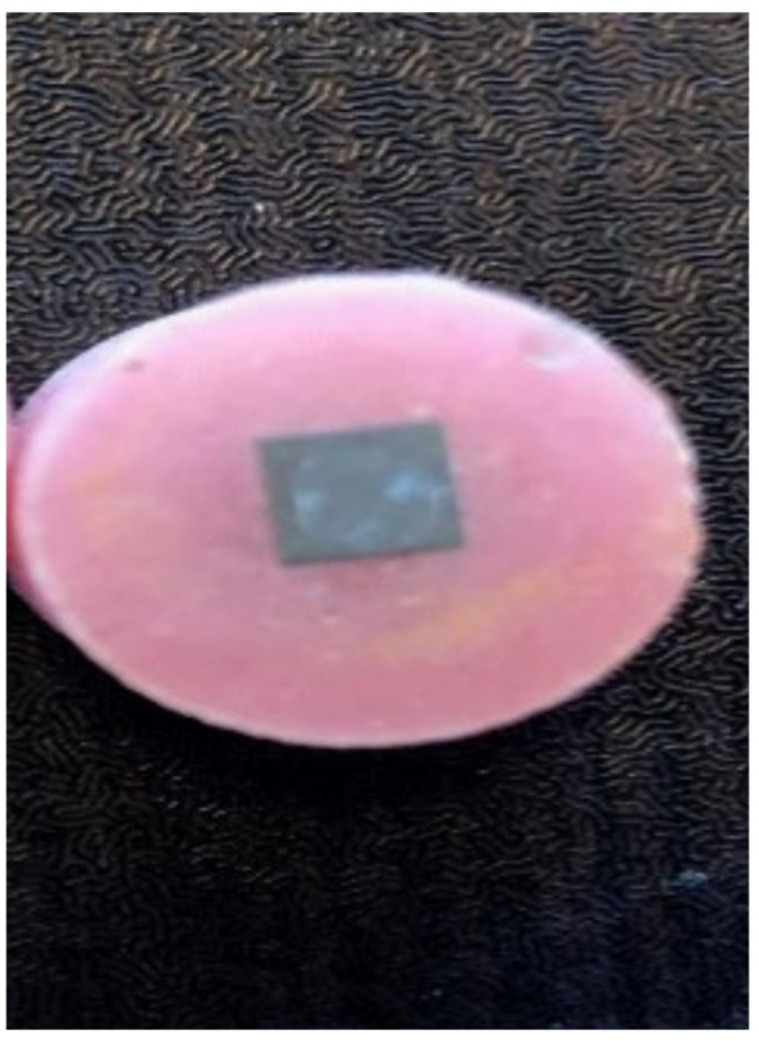
The sample after sandblasting treatment.

Subgroup B: The samples were sandblasted with 250 μm of aluminum oxide at 4 bar of pressure for 10 s in a standardized exposure distance of the sample from the sandblasting nozzle (10 mm); then, Panavia cement was applied. The specimen after sandblasting is demonstrated in Figure 1.

Subgroup C: Samples were immersed in a 35% hydrogen peroxide solution with the treatment of halogen light (Ivoclar-Vivadent AG, Schaan, Liechtenstein) irradiation for 160 s; then, Panavia cement was applied.

In regard to the application of Panavia V5, Clearfil Ceramic Primer (Kuraray Noritake Dental, Tokyo, Japan) was initially applied to the surface of cp-Ti by the usage of a microbrush. Thereafter, the Tygon cylinder with the settled dimensions (1.0 mm internal diameter and 1.0 mm thickness) was filled with Panavia V5. Once the excess of cement was carefully removed with a blade, the cylinders were light-cured for 40 s through a light curing device (Bluephase C8, Ivoclar Vivadent, Schaan, Liechtenstein).

The 45 specimens of the MHA cement group were randomly divided into three subgroups (*n* = 15) as the following:

Subgroup D: The samples were only polished without any other surface treatment to be subjected to the MHA cement.

Subgroup E: The samples were sandblasted with aluminum oxide at 4 bar of pressure for 10 s in a standardized exposure distance of the sample from the sandblasting nozzle (10 mm); then, the MHA cement was applied.

Subgroup F: Samples were immersed in a 35% hydrogen peroxide solution with halogen light (Ivoclar-Vivadent AG, Schaan, Liechtenstein) irradiation treatment for 160 s; then, MHA cement was applied.

Regarding the application of MHA, Monobond Plus (Ivoclar Vivadent AG, Liechtenstein) was initially applied to the surface of cp-Ti by the usage of a microbrush. Then, Tygon tubes with a thickness of 1 mm and diameter of 1 mm were placed on each specimen. Multilink Hybrid Abutment cement (Ivoclar Vivadent, Schaan, Liechtenstein) was applied through a syringe into the Tygon tubes, and the cylinder samples were left for autopolymerization for 10 min.

In the following, the specimens were stored in distilled water at 37 °C for 24 h, and then the Tygon tubes were carefully removed with a scalpel blade. Lastly, the specimens were placed in a thermal cycling machine for 5000 rounds of thermal cycling at 5 °C and 55 °C with a dwell time of 15 s. The 5000 cycles of thermal cycling were equivalent to approximately six months of clinical service [13,14].

**Figure 2:**
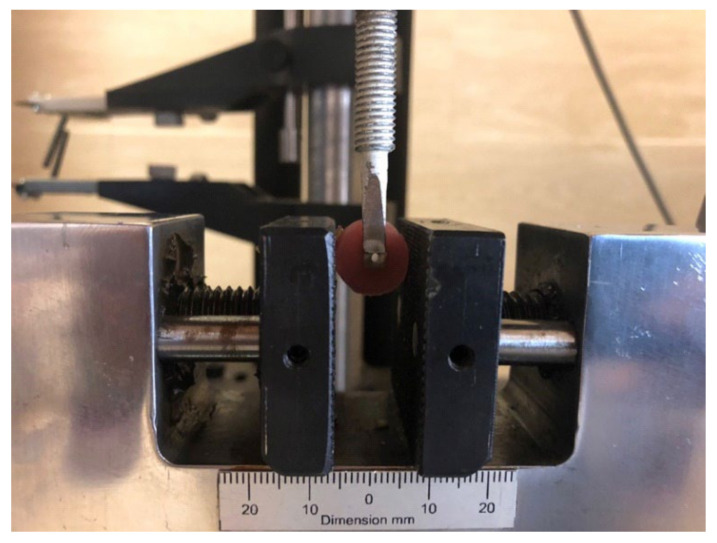
SBS test by using a mechanical universal testing machine.

A mechanical universal testing machine (STM20, SANTAM, Tehran, Iran) was used to measure the microshear bond strength of cement to cp-Ti. The samples were subjected to shear stress at a crosshead speed of 1 mm/min up to the state of fracture (Figure 2). In order to calculate the SBS in megapascals (MPa), the load at failure point (Newton) was divided by the surface area of metal-cement bonding (mm^2^).

Subsequent to the SBS test, the fractured sites were evaluated through a stereomicroscope (Dino lite Pro, Anmo Electro nics Corp, New Taipei City, Taiwan) under a magnification of ×20, while the fracture modes were classified as the following:

1. Cohesive fracture: Fracture within the resin cement.
2. Adhesive fracture: Fracture at resin cement–cp-Ti interface.
3. Mixed fracture: A combination of adhesive and cohesive fractures.

## 3. Results

**Figure 3:**
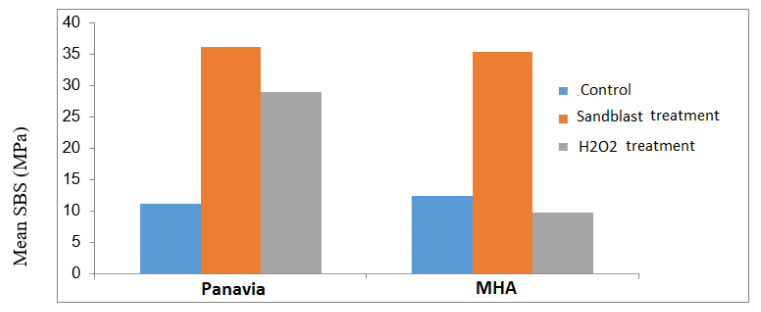
Mean SBS of the Panavia and MHA cements in different surface treatments.

Descriptive statistics for the SBS of the six subgroups are shown in Table 2 and Figure 3. The results of the ANOVA test revealed significant differences between the three subgroups of each group (*p* < 0.001).

The Tukey post hoc test showed that the SBS of subgroup B (sandblast treatment) was significantly higher than that of subgroup C (H_2_O_2_ treatment) (*p* < 0.05), while the SBS of subgroup C was significantly higher than that of subgroup A (control) (*p* < 0.001).

In regard to the MHA group, the Tukey post hoc test indicated that the SBS of subgroup F (H_2_O_2_ treatment) was significantly lower than that of subgroup E (sandblast treatment) (*p* < 0.001), whereas there were no significant differences between the SBS of subgroups D (control) and F (*p* = 0.35) (Table 3).

According to the comparison outcomes of Panavia and MHA cements, the independent samples t-test was indicative of a lack of any significant differences between the subgroups of A and D (*p* = 0.34), as well as the subgroups of B and E (*p* = 0.67). However, a significant difference was observed between the two subgroups of C and F (*p* < 0.001).

The main type of fracture in all of the subgroups was detected to be in adhesive fracture mode. Although the cohesive fracture mode was observed in the case of the Panavia group (subgroups of A and C), there were no signs of cohesive failure in any of the MHA subgroups (Table 4).

## 4. Discussion

The results of the present study indicated that among the applied cements, the sandblasting group obtained the highest SBS when compared to the other two groups. This observation may be attributed to sandblasting with alumina, which causes an improvement in micromechanical roughening of the surface and allows the alumina particles to remain embedded on the surface of cp-Ti [15,16,17]. In other words, the existing alumina particles on metal surfaces have an effective role throughout the bonding mechanism created by bonding systems with functional monomers [16]. According to the results of previous studies on sandblasting treatment, the particle size of 250 μm for alumina particles is favorable for resin penetration and consequently lead to the obtaining of a higher SBS when compared to that of 50 μm particles. In this regard, we considered the application of 250 μm alumina particles for our study [18,19].

Moreover, although H_2_O_2_ treatment caused a higher SBS in the Panavia group than the control group, this difference was not observed in the MHA group between the H_2_O_2_ treatment and control groups.

According to the finding of Yoshida et al. [12], the highest cement shear bond strength was achieved in the case of cp-Ti treated with 34.5% H_2_O_2_ and halogen for 160 s, which was approximately 14 times greater than that of the untreated control cp-Ti plates. However, in this study, the Panavia cement SBS of cp-Ti treated with 35% hydrogen peroxide solution and halogen light irradiation treatment for 160 s was significantly higher than that of the control group. It is believed that the application of a halogen lamp can accelerate the Fenton reaction and significantly shorten the effective procedure of H_2_O_2_ treatment [12].

The performance of H_2_O_2_ treatment can result in increasing the surface roughness, oxide layer thickness, porosity, and, finally, adhesive strength [20]. The differences between the chemistry of the cements could play an important role in differences in the SBS between and within groups. MHA is a dimethacrylate- and HEMA-based adhesive cement system; however, the Panavia V5 cement system includes primers containing phosphate monomers (MDP) [21]. The phosphoric acid group of MDP has chemical affinity to the aluminum oxide particles arrested on the titanium surface [19] and, consequently, helps to improve the SBS. In Panavia, the oxide layer that is enhanced by H_2_O_2_ treatment can improve the bonding between MDP and the titanium surface and increase the SBS [22]. In MHA, the interference of oxidizing agents, such as H_2_O_2_, on the polymerization of HEMA (hydroxyethyl methacrylate) and dimethyl methacrylate may prevent the occurrence of any enhancement in the SBS of MHA group.

Our lack of data on oral conditions is due to performing an in vitro evaluation.

According to the results of the present study, adhesive fracture was the predominant failure mode with respect to the fact that resin cement monomers contain many carbon-carbon double bond units, which produce a high degree of matrix cross-linking and, consequently, high mechanical properties [14].

Maltzahn et al. [23] investigated the effect of different pretreatment conditions on the retention forces between copings composed of zirconia or lithium silicate ceramic and titanium bases of two-part abutments. Their results determined the higher effectiveness of surface mechanical pretreatment by alumina sandblasting compared to the chemical one.

In addition, the report of Guilherme et al. [24] demonstrated that SBS can be enhanced through the performance of treatments with alumina airborne-particle abrasion, alone or etching with 95% HF, for the duration of 30 s.

Pitta et al. [25] provided an evaluation of the effect of different airborne-particle abrasion (APA) methods of the Ti-base surface on the stability of the bonded interface and retention forces between the titanium bases and lithium disilicate crowns after thermomechanical aging. Their results revealed that airborne-particle abrasion of the titanium surface caused an increase in the bond stability and retention forces between the Ti-base and the respective crown, especially upon the usage of alumina particles.

The results of a study conducted by Nakhaei et al. [14], which examined the SBS of different cements to cp-Ti, demonstrated Panavia F.2 as the best choice for bonding to cp-Ti. In another study, Nakhaei et al. [26] investigated the SBS of different bonding protocols to commercially pure titanium (cp-Ti) through the use of two universal adhesives, including Scotchbond Universal (SU; 3M ESPE, St. Paul, MN, USA) and G-Premio Bond (GC Corporation, Tokyo, Japan) and Alloy Primer (AP; Kuraray). Their results confirmed that the application of AP, followed by SU, was the most superior bonding to cp-Ti and contained the ability to endure the limited thermal aging.

Our lack of data on oral conditions due to performing an in vitro evaluation is the major limitation of our study. Therefore, future studies could provide more detailed results by closely mirroring the in vivo situations.

## 5. Conclusions

In conclusion, sandblasting treatment proved to be capable of improving the bond strength of the titanium surface in both cases of Panavia V5 and MHA cements. However, the performance of H_2_O_2_ treatment enhanced the bond strength of Panavia V5 cement without causing any positive effects on the bond strength of MHA cement.

## Figures and Tables

**Table 1:** The chemical composition of the cements.

Cement	Composition
Panavia V5	Bisphenol A diglycidylmethacrylate (Bis-GMA), Triethyleneglycol dimethacrylate (TEGDMA), Hydrophobic aromatic dimethacrylate, Hydrophilic aliphatic dimethacrylate, Initiators Accelerators, Silanated barium glass filler, Silanated fluoroalminosilicate glass filler, Colloidal silica Bisphenol A, Silanated alminum oxide filler, dl-Camphorquinone, Pigments
MultiLink Hybrid-Abutment (MHA)	Dimethacrylate, HEMA, fillers (barium glass, ytterbium trifluoride, spheroid mixed oxides, titanium dioxide)

**Table 2:** Mean and standard deviation of the SBS in six different groups.

Subgroups	*n*	Mean (MPa)	Standard Deviation (MPa)
A	15	11.06	2.99
B	15	36.13	5.54
C	15	28.86	7.37
D	15	18.55	3.30
E	15	35.28	4.33
F	15	9.7	5.37

**Table 3:** Post hoc Tukey’s multiple comparison test between groups for SBS.

Subgroup	Subgroup	*p*-Value
A	B	*p* < 0.001
A	C	*p* < 0.001
B	C	*p* = 0.01
D	E	*p* < 0.001
D	F	0.35
E	F	*p* < 0.001

**Table 4:** Frequency distribution of type of fracture for six groups.

Subgroups	Failure Mode		
	Adhesive (%)	Mixed (%)	Cohesive (%)
A	66.6	20	13.3
B	80	20	0
C	66.6	26.6	6.6
D	93.3	6.6	0
F	53.3	46.6	0
F	80	20	0

## Data Availability

Data is contained within the article.
